# ELectrophysiological mechanisms underlying the Inhibitory CArdiac syncope without asystolic significant pause: Therapeutic and prognostic implications. The ELICA randomized trial

**DOI:** 10.1097/MD.0000000000011757

**Published:** 2018-08-03

**Authors:** Celestino Sardu, Raffaele Marfella, Gianluca Testa, Matteo Santamaria, Cosimo Sacra, Alfredo Ranauro, Giuseppe Paolisso, Maria Rosaria Rizzo, Michelangela Barbieri

**Affiliations:** a Department of Medical, Surgical, Neurological, Metabolic and Aging Sciences, University of Campania “Luigi Vanvitelli”, Naples; b Department of Medicine and Health Sciences, University of Molise; c Cardiovascular and Arrhythmias Department, John Paul II Research and Care Foundation, Campobasso, Italy.

**Keywords:** catheter ablation, devices implants, electrophysiological study, syncope, syncope recurrence, tilt test, vasovagal syncope

## Abstract

**Background:**

The aim of this study was to investigate syncope recurrence in patients with a 2A cardioinhibitory response to the head up tilt testing (HUT).

**Methods:**

In this study, we enrolled 72 consecutive patients affected by syncope with cardioinhibitory response without asystolic significant pause to HUT (2A type). In these patients, we randomly performed electrophysiological study (ES). In case of sino-atrial node, atrio-ventricular node dysfunction, and sustained arrhythmias induction, the ES resulted positive. ES was positive in 9 patients (group A), then treated by catheter ablation, and/or by devices implants. Otherwise, ES resulted negative (group B), and these patients did not receive an interventional treatment. However, after ES, we evaluated syncope recurrence during 360 days follow-up.

**Results:**

There was a lower statistical significant syncope recurrence at follow-up, comparing group A to group B of patients [n of events 9 (40.9%) vs 8 (57.1%), *P* < .05]. At multivariate analysis, ES result was the only factor predicting syncope recurrence at follow-up (hazard ratio = 27.63, 95% confidence interval = 1.02–54.24, *P* < .005).

**Conclusion:**

The positivity to ES study, and successful interventional therapies may reduce the burden of syncope recurrence at 360 days follow-up in 2A HUT subjects.

Clinical trial number: NCT02861274.

## 1 Introduction

Syncope is a transient loss of consciousness due to transient global cerebral hypoperfusion, and characterized by rapid onset, short duration, and spontaneous complete recovery.^[1]^ Syncope is frequent in general population, increasing with age, and related to inappropriate response of the autonomic nervous system, by an excessive vagal tone, and sympathetic tone withdrawal.^[1,2]^ Therefore, syncope is an emergency setting, with an increasing lifetime cumulative incidence.^[3]^ Syncope may be preceded by warning symptoms, which are named prodromes.^[1]^ Prodromes are lightheadedness, nausea, sweating, weakness, and visual disturbances, and they may differ in terms of first presentation, modality of presentation, and duration.^[1]^ On the contrary, syncope may occur without prodromes, and there may be prodromes not followed by syncope.^[1]^ Moreover, the syncope recurrence is a relevant clinical problem, with a great impact on quality of life.^[4,5]^ In this setting, authors have proposed head up tilt testing (HUT) to stage and diagnose the different types of syncope.^[1,2]^ Indeed, the diagnostic power of HUT is to lead to blood pooling, decreasing the venous return due to orthostatic stress, and triggering the vaso-vagal reflex.^[1,2]^ Moreover, HUT may reproduce the different cardioreflex responses such as the cardioinhibitory response.^[1,2]^ The cardioinhibitory syncope is divided into 2 forms: a form without significant asystolic pause (2A type), and a form with significant asystolic pause (2B type).^[6]^ In detail, in 2A HUT response, the blood pressure falling, and the heart rate falling leading to loss of conscious, and their temporal correlation are not associated with significant asystolic pause.^[1]^ Moreover, this pathogenic mechanism differentiates specifically the 2A HUT response versus 2B HUT response, and from the other HUT responses.^[1]^ Furthermore, if cardiac pacing may be indicated in patients with 2B type HUT response, it is not indicated in the 2A type HUT patients.^[1]^ Moreover, in this study, we focused on cardioinhibitory 2A syncope. The 2A syncope may be due to complexes and underinvestigated mechanisms, and triggering factors. In this setting, we may speculate that “arrhythmic triggers” may work as factors leading to the clinical event. To date, at moment, there are no studies investigating the underlying cardiac rhythm disorders in cardioinhibitory 2A syncope patients without structural heart disease. Consequently, because of its pathogenesis, the lack of therapeutic options, and the higher recurrence rate, the cardioinhibitory 2A syncope may represent a challenging emergency setting. Therefore, our study hypothesis was that, in a proportion of patients presenting with syncope, and 2A HUT response, the symptoms may be due, and/or increased by arrhythmic triggers. Moreover, in these patients, the cardioinhibitory response without asystolic pause may be an adaptive mechanism of an arrhythmic stress condition. Indeed, in these patients, the electrophysiological study (ES) may be used to diagnose underinvestigated conduction disturbance and/or arrhythmias, which can be treated by an interventional approach. Moreover, in this study, we wished to determine whether to use ES to diagnose arrhythmic disturbances in 2A HUT subjects, to use ES to treat arrhythmias, and to prevent syncope recurrence in patients with cardioreflex syncope, to report in 2A HUT subjects after a ES (positive v/s negative result) the time of the first syncope recurrence, to evidence the presence of factors predictive of others syncope events in investigated patients, and to evaluate ES impact on clinical outcomes during a long-term follow-up.

## 2 Materials and methods

**Figure 1:**
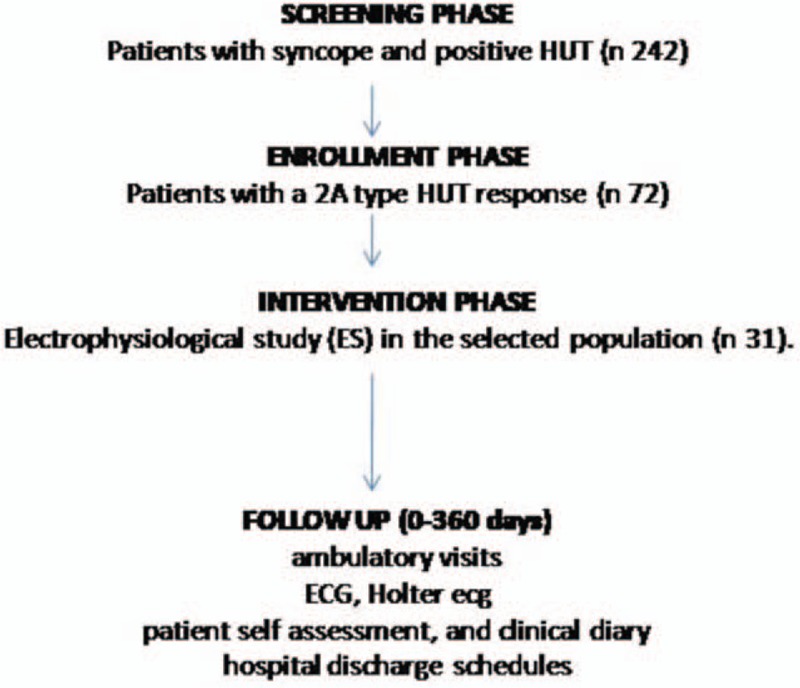
In this figure, there is a representation of study phases. SCREENING PHASE is first phase of the study, to screen in all population of patients affected by syncope, the patients with a positive response to head up tilt test (HUT). The patients with a positive HUT were 242. The second phase of the study, the ENROLLMENT PHASE, has been conducted to enroll all patients with a positive 2 A type HUT response. These patients were affected by syncope associated with a cardioinhibitory response, in absence of asystolic pause. As described in the text, these patients were randomly treated by electrophysiological study (ES). This was the third phase of the study, the Intervention phase (n = 31 patients). After the ES, patients were followed up for 12 months (ambulatory visits, ECG, Holter ECG, patient self-assessment, and clinical diary, hospital discharge schedules for syncope recurrence events).

In a prospective multicenter randomized study, conducted from January 2013 to January 2015, at Catholic University of Sacred Heart, Campobasso, Italy, at John Paul II Research and Care Foundation, Campobasso, Italy, and at Second University Study of Naples, Italy, we studied a population of 242 patients with syncope and positive HUT. In these patients, we performed an HUT, as suggested by international guidelines.^[1]^ Eligible patients in the study had to follow these inclusion criteria: aged more than 18 years, absence of cardiac structural diseases, patients with an indication to receive a HUT, patients with a 2 A type HUT response, and patients with an indication to receive a diagnostic ES after a 2 A type HUT diagnose. However, we performed an ES in patients with suspected intermittent bradycardia, bundle branch block, and suspected tachycardia. From this study, patients with a known history of atrial, supraventricular and ventricular arrhythmias, and depression of the left ventricle ejection fraction were excluded. Moreover, we screened a population of 72 patients with a vasovagal cardiac syncope without significant asystolia (2 A type HUT). We analyzed the patients’ clinical prodromes, and we divided these patients in 2A HUT with prodromes versus 2A HUT without prodromes (Fig. 1). The selected patients randomly received an ES in a parallel study, in an allocation 1:1. We used a computer programming code for treatment randomization. However, we screened 36 patients for ES, and we performed ES in 31 patients. Patients excluded to perform an ES were n = 1 refused to receive an ES, n = 1 did not sign informed consent to perform the study, and n = 3 did not have indications to receive an ES (Fig. 1). The follow-up duration was 12 months. The 12 months period was selected as a reasonably long period of observation, and by definition, patients had to have had at least 1 recurrence of syncope to qualify for the study. All patients were informed of the nature of the study and provided written consent. The study was conducted in accordance with the Declaration of Helsinki. The protocol was approved by the Ethics Committees of all participating institutions. The clinical trial is registered in “ClinicalTrials.gov,” registration number: NCT02861274, registration date August 10, 2016.

### 2.1 Study endpoints

As study endpoints, we evaluated syncope recurrence at follow-up in patients affected by cardioinhibitory syncope without asystolic pause, cardiac deaths, and all cause deaths.

### 2.2 Patients monitoring

These patients were scheduled for an in-office follow-up visit 14 days after clinical discharge, and after 1, 3, 6, and 12 months by the treating physician. These patients were monitored by ambulatory follow-up. All the patients gave their written informed to participate in the trail. Clinical evaluation included physical examination, vital signs, and review of adverse events. A fasting blood (at least 12 hours from last meal) was performed for glycemia, lipid profile [total cholesterol (TC), triglycerides, high-density lipoprotein-cholesterol (HDL-C), and low-density lipoprotein-cholesterol (LDL-C)], and C-reactive protein (CRP) at every visit. Syncope recurrence and other clinical events were collected during patients interview, visits, and by hospital discharge schedules.

### 2.3 Head Up Tilt Test (HUT)

The HUT was always performed in the morning in a quiet room with the lights slightly dimmed, after overnight fasting. The procedure was carried out using a motorized tilt table with foot support according to European Society of Cardiology Syncope Guidelines.^[1]^ After a 5-minute supine control phase, patients were moved to the 60° upright position for a maximum duration of 45 minutes or until syncope developed.^[1]^ At 20 minutes, 400 μg of nitroglycerin spray was administered sublingually.^[1,6]^ At the time of syncope, patients were immediately tilted back to the horizontal position.^[1,6]^ The HUT was considered positive if syncope developed in association with hypotension, bradycardia, or both.^[1,6]^ In the 2A type HUT, syncope developed in association with hypotension, and bradycardia, in absence of significant asystolic pause.^[1,6]^

### 2.4 Electrophysiological study (ES)

After right femoral vein puncture, a 4-electrode electrophysiology catheter was placed in high right atrium (HRA), and a second 4-electrode electrophysiology catheter was placed in right ventricle apex (RVA), and his region (HIS) (Josephson curve diagnostic catheters; St Jude Medical, Minneapolis, MN). These catheters were used for HRA, RVA, and HIS potentials registration, and for pacing maneuvers to study cardiac conduction (Sino Atrial Node function, anterograde and retrograde atrioventricular node function, and arrhythmias induction).^[7]^ After baseline assessment, the ES was repeated during isoproterenol via intravenous drip, then given to increase the heart rate up to 120 bpm. Burst atrial pacing (at a circle length from 300 to 200 ms) and atrial programmed coupled stimulation (at drive cycle length 600 and 400 ms) were performed from HRA to observe whether there was atrial tachycardia, atrial flutter, or atrial fibrillation episodes.^[7]^ Similarly, the same protocol was performed placing a catheter in RVA to induce ventricular arrhythmias.^[7]^ In case of sinoatrial node, atrioventricular node dysfunction, and sustained arrhythmias induction, the ES resulted positive. On the contrary, it was classified as negative. However, conduction disturbance at baseline were identified by the evidence of prolonged supra-Hisian (AH interval normal value 50–120 ms) and infra-Hisian conduction (HV interval normal value <55 ms). During atrial programmed pacing maneuvers, we identified the sinoatrial node dysfunction and the atrioventricular node dysfunction. The sino-atrial node dysfunction was identified, during 60” of continuous atrial pacing at 600 and 400 ms, by the evidence of a prolonged and abnormal sinus node recovery time (SNRT) as >1.6 or 2 seconds for SNRT, or >525 ms for corrected sinus node recovery time (CSNRT).^[8]^ Atrioventricular node dysfunction was diagnosed by the evidence, during a programmed decremental and coupled atrial pacing protocol, of an abnormal Wencheback point (WP normal values <400 ms), and of an abnormal effective refractory period of atrioventricular node (ERPAVN normal values 280–420 ms).^[8]^ Sustained arrhythmias induction was defined as the induction of a prolonged arrhythmic event (> 30” duration) during a programmed atrial and/or ventricular pacing protocol.^[8]^ This arrhythmia may originate from atrial chamber, atrioventricular node, concealed accessory pathways, and/or from ventricular chambers.^[8]^ In the case of positive ES, an interventional treatment was performed as follows: pacemaker implant in case of pathological sinoatrial node and atrioventricular node dysfunction; internal cardioverter defibrillator implant in case of sustained ventricular arrhythmias; trans catheter ablation in case of sustained supraventricular, and atrial tachy-arrhythmias. In case of negative ES result (n = 22), patients received drug therapy as recommended: beta blockers (n = 4), fludocortisone (n = 2), and midodrine (n = 16).

### 2.5 Statistical methods

All data were analyzed by 2 different physicians, and the patients divided before in patients with prodromes (n = 22) versus patients without prodromes (n = 14), and during follow-up visits and controls in patients with ES-positive result (n = 9) versus in patients with ES-negative result (n = 22). Normally distributed variables were tested by 2-tailed Student *t* test for paired or unpaired data, as appropriate, or by 1-way analysis of variance (ANOVA) for more than 2 independent groups of data. The categorical variables were compared by Chi-square or Fisher exact test where appropriate. The statistical significance was set at *P* < .05 (2-sided tests), and for multiple testing, we used a statistical significance of *P* < .05. A multivariable logistic regression analysis was conducted. Among all risk factors and all clinical and angiographic parameters evaluated (age, sex, resting heart rate, systolic and diastolic blood pressure, etc), only the variables presenting a *P* value ≤.25 at univariable analysis were included in the model. The stepwise method with backward elimination was used and odds ratios (ORs) with 95% confidence intervals (95% CIs) were calculated. Sample size calculation was done using a computer software by an established CI, and a confidence level of 95% for the study population, resulting in the study population of 69 patients. The power of the study was assessed using π = 0.80 as a standard for adequacy. Statistical analysis was performed using the SPSS software package for Windows 17.0 (SPSS Inc., Chicago, IL). Statistical analysis was performed using the SPSS software package for Windows 17.0 (SPSS Inc., Chicago, IL).

## 3 Results

**Figure 2:**
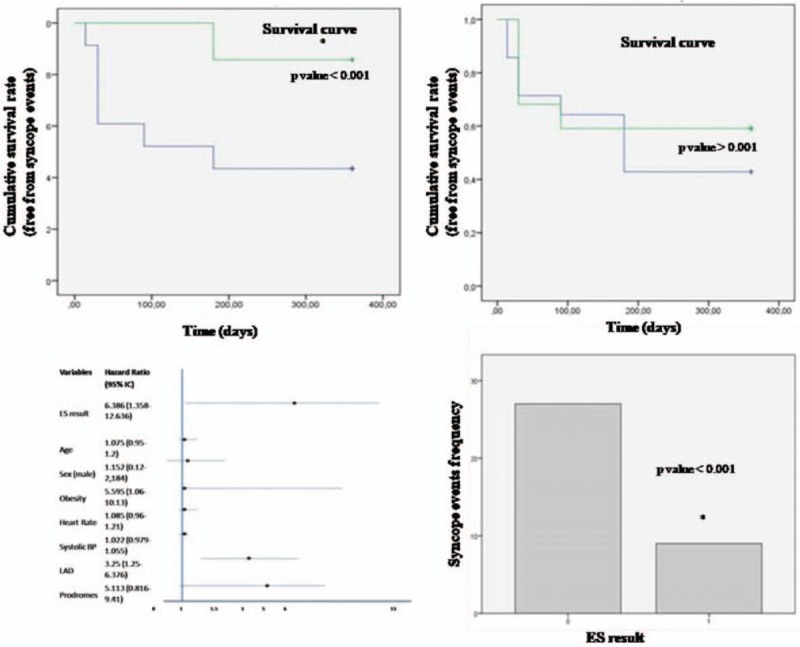
In this figure, upper part survival curves “free from syncope recurrence” at 360 days follow-up. In the left and right upper parts, on y axis representation of syncope recurrence events. In the x axis, representation of time in days. At Kaplan–Meier analysis, at follow-up, there was a lower rate of syncope recurrence events in patients with a positive result (green color) at electrophysiological study (ES) than patients with a negative result (blue color) (*P* < .001). This value was marked by symbol ^∗^. In right upper part, there was a lower but not significant syncope recurrence rate comparing patients with prodromes (green color) versus patients without prodromes (blue color) (*P* > .001). In lower inferior figure part on the left, representation of univariate analysis result to predict syncope recurrence at follow-up by Cox regression analysis. On the right inferior part, the frequency of syncope recurrence in patients with a negative ES result (left part) marked with number 0, and positive ES result marked with number 1 (right part). This statistical significant event (*P* < .05) was marked with the symbol^∗^.

Clinical characteristics of general population screened from our database are summarized in Table 1. The clinical history was identical and nondiscriminatory in all patients with a VASIS 2A response. After diagnosis of 2A type HUT, 72 selected patients randomly received an ES. We reported clinical characteristics in Table 2, and ES results of the 72 enrolled patients divided into patients with prodromes (n = 22) versus patients without prodromes (n = 14). About the patients with a positive ES result, 5 of 22 patients (22.7%) had prodromes versus 4/10 (40%) patients who did not have prodromes. The 9 patients with a positive ES result were treated by catheter ablation (n = 2) and/or by devices implant (n = 6 pacemakers, 1 implantable cardioverter defibrillator). Twenty patients with a negative ES were treated by a conventional drug treatment for syncope recurrence. All patients were followed ambulatory as described before in the text. The recurrence of acute, and/or delayed syncope-free survival, from 14 to 360 days follow-up, in ES-positive result group of patients was lower than the group of patients with a negative ES result (log-rank *P* < .05; Fig. 2). At multivariate analysis by Cox regression, we tested obesity [hazard ratio (HR) = 5.595, 95% CI = 1.06–10.13, *P* < .05], systolic blood pressure (HR = 1.022, 95% CI = 0.979–1.055, *P* > .05), left atrium dilatation (HR = 3.25, 95% CI = 1.25–6.376, *P* < .05), prodromes (HR = 5.113, 95% CI = 0.816–9.41, *P* > .05), and negative result to the ES (HR = 6.386, 95% CI = 1.358–12.636, *P* < .005), as factors to predict the event of syncope recurrence at long-term follow-up (Table 3, Fig. 2). At multivariate analysis, a negative ES result was the only factor predicting syncope recurrence at follow-up (HR = 2.763, 95% CI = 1.02–5.424, *P* < .005; Table 3). No cases of cardiac death and overall causes mortality were reported in the present study.

**Table 1:**
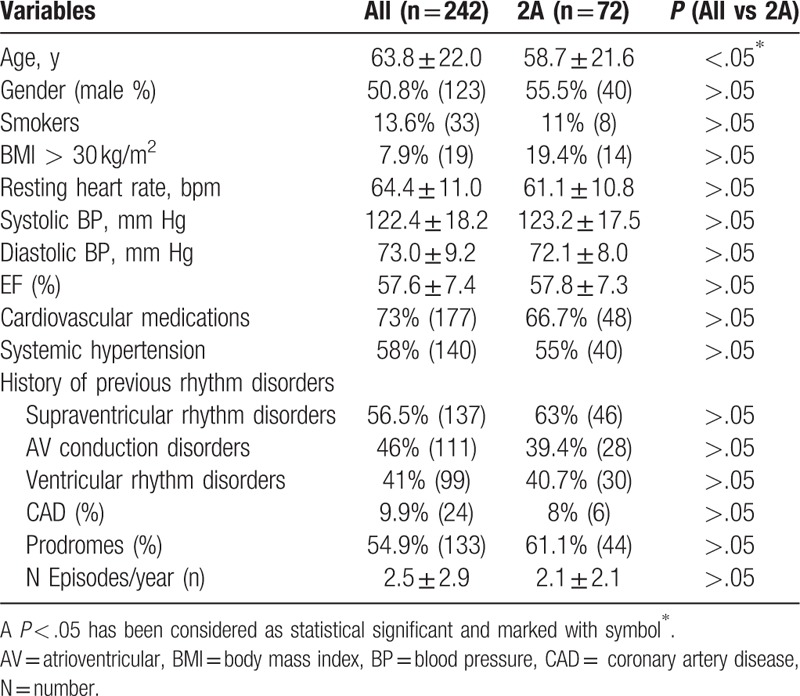
Baseline characteristics of all 242 patients studied with a positive tilt-test (HUT), and of type 2A HUT subgroup of patients (n = 72).

**Table 2:**
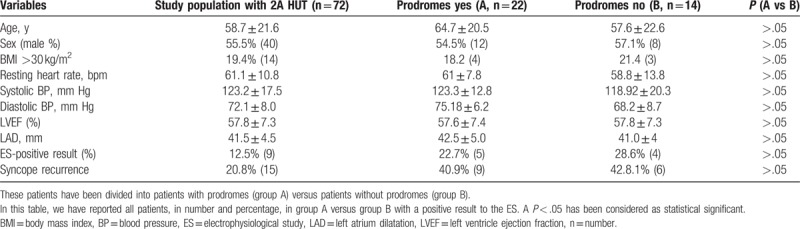
Baseline characteristics of all 36 patients studied with a positive 2A type tilt-test (HUT).

**Table 3:**
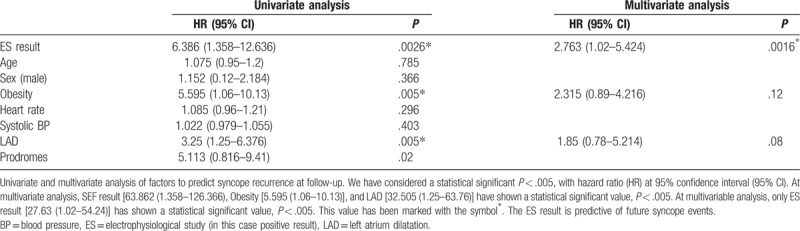
Multivariate cox regression analysis for parameters associated with study endpoint.

## 4 Discussion

Syncope recurrence is a relevant clinical problem.^[7]^ In our study, not differently from other authors,^[9]^ we analyzed prodromes as warning symptoms, and as predictors of syncope recurrence. In fact, prodromes may alert a warning condition during a syncope event.^[10]^ Consequently, this may lead patients to start maneuvers to reduce and to avoid the clinical event.^[10]^ To date, these maneuvers are a risk-free and an effective method to avoid syncope, and are a first-line treatment of vasovagal syncope.^[10]^ On the contrary, patients without prodromes may directly present syncope, without the time to avoid the event.^[11]^ Different mechanisms may explain the absence of prodromes during a syncope event, such as the alterations of cardiac ionic channels activity, and cardiac conductions properties.^[11]^ These alterations may be under hand, and not detectable by routine clinical assessment.^[11]^ A part of this, in our study population, we observed that, the presence of prodromes did not affect the prognosis after the syncope event. We may speculate that, the cardioreflex 2A syncope may be due to other complex pathological triggering mechanisms, such as alterations in cardiac ionic channels activity, and cardiac conductions properties.^[11]^ However, all these pathological aspects may differentiate the 2A syncope as compared with other forms of cardioreflex syncope. In addition, in 2A HUT patients, we found that, at multivariate analysis, the negative ES result was predictive of syncope recurrence (HR = 2.763, 95% CI = 1.02–5.424, *P* < .005; Fig. 2). Numerous observations may explain this study result. It looks comprehensible that ES may diagnose an arrhythmic disturbance and/or a cardiac conduction dysfunction related to the syncope event. However, the arrhythmic disorder may trigger and/or enhance the syncope event. Consequently, the underlying arrhythmic disorder may be underinvestigated and under treated during the syncope event, and this may consequently affect syncope recurrence. In this context, independently from vagal tone alterations and its impact on syncope pathogenesis, ES may diagnose an arrhythmic condition and/or a cardiac conduction dysfunction, which are not fully investigated by ordinary clinical examination.^[8]^ In fact, the ES may study conduction heart system, and arrhythmic disorders at baseline, and during stress conditions.^[8]^ Consequently, ES may drive the physicians to a curative treatment by catheter ablation (sustained tachy-arrhythmias), by implant of pacemakers (brady-arrhythmias, significant sinus node, and atrial-ventricular node dysfunction), and/or by implant of implantable cardioverter defibrillators (sustained ventricular arrhythmias).^[8]^ These interventions may treat the arrhythmic condition triggering the syncope event,^[8]^ and this may reduce and/or avoid the syncope recurrence. On the contrary, a negative ES result may not clarify the arrhythmic pathogenic mechanism related to the syncope event. Subsequently, a negative ES result does not lead to interventions and treatments to avoid syncope recurrence.^[12]^ In fact, in our study, patients with a negative ES result reported higher syncope recurrence than patients with a positive ES result. Consequently, this pushes us to speculate that the syncope event and its recurrence may be related to subclinical alterations of cardiac conduction properties, and/or arrhythmic abnormalities in patients with a 2A syncope. However, the association between cardiac conduction properties/arrhythmic abnormalities and syncope as first event and recurrence event in 2A syncope patients is an actual and relevant object of study.^[12]^ In this setting, recently, authors suggested the implantation of continuous monitoring devices to detect subclinical arrhythmias linked to unexplained syncope events.^[13]^ These devices collected information about patients’ symptoms, and arrhythmias linked to syncope of unexplained nature, by the continuous registration of heart rate.^[13]^ On the contrary, these devices cannot treat the syncope event, and they cannot avoid syncope recurrence at follow-up.^[13]^ Similarly, also, new drugs did not lead to a significant reduction of syncope recurrence.^[14]^ However, the unexplained syncope event, and its recurrence in 2A HUT patients are still a relevant clinical problem, and this represented the central idea of our study investigation. In fact, our investigation focused on the study of syncope pathogenesis, and of its recurrence in 2A cardioreflex syncope, such as a different form and a clinical distinct entity of syncope as compared to all the other. Not far from our study hypothesis, recently, authors focused on arrhythmic disorders as syncope triggers, proposing the 4-week external ECG monitoring as first-line tool in the diagnostic work-up of syncope.^[15]^ They suggested that, the early recording, the history of arrhythmias, and frequent previous events, increased in statistical significant way the likelihood of diagnostic events during the 4-week external ECG monitoring.^[15]^ In our study, as first ES diagnosed arrhythmic and cardiac conduction abnormalities, and secondary ES drove physicians toward curative treatments during the same hospital admission for the syncope event.^[16]^ Consequently, the treated patients showed benefits in terms of syncope recurrence at follow-up. In this way, we may speculate to have modified an arrhythmic disorder triggering the syncope event, and then associated with syncope recurrence. Differently, the negative ES result may define a class of patients more difficult to treat, and related to worse prognosis.^[17]^ At moment, we do not know the clear association between subclinical arrhythmic disorders, and cardioreflex syncope, but we may report the utility of ES to reduce syncope recurrence in selected 2A cardioreflex syncope patients. Therefore, jointly with other authors’ suggestions,^[18]^ we may propose new diagnostic approaches for the diagnosis, and for the treatment of cardioreflex syncope recurrence. In this setting, new interventional treatments have been proposed to reduce syncope recurrence by the control of the excessive vagal activity. Regarding these new treatments, we may mention the radiofrequency catheter ablation of the areas related to the 3 main autonomic ganglia around the heart.^[19]^ This treatment is a lesser-known technique for management of patients with excessive vagal activation, and it has been proposed as an alternative and safe strategy to reduce syncope recurrence especially in young patients.^[19]^ Intriguingly, the enhanced parasympathetic tone may cause sinus bradycardia or pauses, and transient or permanent atrioventricular block, with resultant vasovagal syncope in patients, that may be highly symptomatic and refractory to the conventional therapies, and that may require cardiac pacemaker implantation.^[20]^ However, the radiofrequency catheter ablation of main parasympathetic autonomic ganglia around the heart may be applied also in patients affected by brady-arrhythmias.^[20]^ On the contrary, the complicated inclusion criteria, ganglia detection methods, and ablation endpoints do not recommend the routine usage of this ablative procedure.^[20]^

## 5 Conclusion

Syncope recurrence, and the loss of a model to predict, and to treat a future syncope event in patients affected by cardioinhibitory reflex syncope after a positive HUT, is a relevant clinical problem. Therefore, in these patients, the opportunity to detect subclinical arrhythmic conditions linked to the syncope event may help physician to diagnose, to treat, and to reduce syncope recurrence. In our study, we supposed that, an autonomic dysfunction triggered a syncope event,^[1]^ and an arrhythmic condition probably related to a cardiac reflex syncope.^[21,22]^ These arrhythmic abnormalities may be more or less pronounced in the same clinical setting as the cardioreflex syncope event, and detectable by an ES. However, in this study, we focused on 2A HUT patients, and on the ES using to drive to successful interventional therapies, and to reduce syncope recurrence at follow-up in this population. This result may open the field to new diagnostic approaches, and treatments to avoid syncope recurrence in 2A HUT patients. Therefore, according to other authors,^[18–22]^ we may propose ES as new diagnostic approach to reduce cardioreflex syncope recurrence. This treatment may change the historical course of the cardioreflex syncope, reducing syncope recurrence.

### 5.1 Study limitations

In this study, not all centers contributed with the same amounts of patients, but no significant differences were observed among patients enrolled by the different centers. The information provided by patients’ diary was used to categorize for the absence or presence of symptom (syncope or palpitation) at the time of syncope. The events reported in the diary by each patient were referred at physician at each clinic visit. The follow-up was 360 days, and the short-term duration of it may affect long-term clinical outcomes. After HUT and ES, and clinical discharge, patients’ arrhythmias recurrence was detected by surface ECG registration, ECG Holter monitoring, and this may represent another study limitation. In our study, we did not evaluate the plasmatic levels of adenosine, which were reported to be lower in patients affected by paroxysmal atrioventricular block as compared with vaso-vagal syncope.^[19]^ This study population was too small to draw any definitive conclusion on the possible correlation between ES-negative and syncope recurrence findings. The capability of ES to influence therapeutic decisions or to improve clinical outcomes was beyond of the scope of this study, and it remains to be demonstrated by an appropriately designed study.

## Author contributions

A.R. collected data.

Ce.S. designed the study and wrote the main manuscript text.

Co.S. prepared Tables 1–3.

G.P. reviewed the study.

G.T. wrote the main manuscript text.

M.B. wrote the main manuscript text.

M.R.R. reviewed the study.

M.S. prepared Figs. 1 and 2.

R.M. reviewed the study.

**Conceptualization:** Celestino Sardu.

**Data curation:** Celestino Sardu, Alfredo Ranauro, Maria Rosaria Rizzo.

**Formal analysis:** Celestino Sardu.

**Investigation:** Celestino Sardu.

**Methodology:** Celestino Sardu, Gianluca Testa, Michelangela Barbieri.

**Project administration:** Matteo Santamaria, Cosimo Sacra.

**Supervision:** Raffaele Marfella, Giuseppe Paolisso.

**Validation:** Raffaele Marfella.

**Writing – original draft:** Celestino Sardu.

Author name: orcid number.
